# Incubation Temperature, But Not Pequi Oil Supplementation, Affects Methane Production, and the Ruminal Microbiota in a Rumen Simulation Technique (Rusitec) System

**DOI:** 10.3389/fmicb.2017.01076

**Published:** 2017-06-28

**Authors:** Andrea C. Duarte, Devin B. Holman, Trevor W. Alexander, Kerstin Kiri, Gerhard Breves, Alexandre V. Chaves

**Affiliations:** ^1^School of Life and Environmental Sciences, Faculty of Science, The University of Sydney, SydneyNSW, Australia; ^2^Lethbridge Research Centre, Agriculture and Agri-Food Canada, LethbridgeAB, Canada; ^3^Department of Physiology, University of Veterinary MedicineHannover, Germany

**Keywords:** methanogens, fermentation process, greenhouse gas, ruminant nutrition, *in vitro*

## Abstract

Lipid supplementation is a promising strategy for methane mitigation in cattle and has been evaluated using several different lipid sources. However, limited studies have assessed the effect of temperature on methane emissions from cattle and changes in incubation temperature have also not been extensively evaluated. The aim of this study was to evaluate the combined effect of pequi oil (high in unsaturated fatty acids) and incubation temperature on fermentation characteristics and microbial communities using the rumen simulation technique. A completely randomized experiment was conducted over a 28-day period using a Rusitec system. The experiment was divided into four periods of 7 days each, the first of which was a 7-day adaptation period followed by three experimental periods. The two treatments consisted of a control diet (no pequi oil inclusion) and a diet supplemented with pequi oil (1.5 mL/day) which increased the dietary fat content to 6% (dry matter, DM-basis). Three fermenter vessels (i.e., replicates) were allocated to each treatment. In the first experimental period, the incubation temperature was maintained at 39°C, decreased to 35°C in the second experimental period and then increased again to 39°C in the third. Pequi oil was continuously supplemented during the experiment. Microbial communities were assessed using high-throughput sequencing of the archaeal and bacterial 16S rRNA gene. Methane production was reduced by 57% following a 4°C decrease in incubation temperature. Supplementation with pequi oil increased the dietary fat content to 6% (DM-basis) but did not affect methane production. Analysis of the microbiota revealed that decreasing incubation temperature to 35°C affected the archaeal and bacterial diversity and richness of liquid-associated microbes, but lipid supplementation did not change microbial diversity.

## Introduction

Methane (CH_4_) is produced in the rumen by a group of archaea known as methanogens. Methanogens have an unusual metabolism because they are capable of using substrates such as hydrogen, carbon dioxide (CO_2_), formate, methylated C1 compounds and acetate as energy and carbon sources for growth and produce CH_4_ as a major end product (Deppenmeier, 2002). The production of CH_4_ during fermentation in the bovine rumen also results in a loss of energy for the animal. Depending on feed intake as well as diet composition, around 2 to 12% of gross energy intake is converted into CH_4_ (Johnson and Johnson, 1995). Measures to mitigate enteric fermentation would not only reduce emissions, but may also raise animal productivity by increasing digestive efficiency.

A number of studies have reviewed methane mitigation strategies involving the modification of dietary composition or through dietary supplementation with lipids, organic acids, plant components, etc. (Hook et al., 2010). Among documented methane abatement strategies, dietary modification appears to be one of the promising technologies to reduce methane emissions from ruminants (Beauchemin et al., 2008; Martin et al., 2010; Patra, 2013). For example, the addition of fats to ruminant diets is one of the recognized means used to decrease enteric methane emissions (Beauchemin et al., 2008). Previous batch culture incubations in our research group indicated that pequi (*Caryocar brasiliense*) oil used at 1% v/v can reduce CH_4_ production by up to 74% (unpublished data).

Microbial activity in the rumen is known to be affected by dietary additives (Hristov et al., 2013) but information related to the effect of rumen temperature and/or incubation temperature on methane production is scarce. Graham et al. (1959) have shown that CH_4_ production in adult sheep decreased by 20% when the environmental temperature was reduced from 33 to 8°C. Others have also indicated a 30% CH_4_ reduction in cold-adapted sheep (Kennedy and Milligan, 1978) while Rogerson (1960) suggested that the effect of environmental temperatures changes between 20 and 40°C on CH_4_ production in cattle were variable. However, limited research has been conducted on the effect of incubation temperature changes on the ruminal microbiota and on fermentation characteristics. It has been suggested that microbial diversity in the bovine rumen decreases concurrently with the ambient temperature and that some bacteria adapt better to adverse environmental conditions (Romero-Perez et al., 2011), and this may also affect rumen function. Crater and Barboza (2007) demonstrated that the rumen of muskoxen (*Ovibos moschatus*) reached minimal temperatures of 31.4°C, 35.7°C, 32.5°C in early, middle and late winter, respectively. According to Crater and Barboza (2007) minimal rumen temperatures were much more variable than maxima. Ruminal cooling was often rapid (–6.24°C/min), dropping to 27.6°C within a few minutes.

We hypothesized that the inclusion of pequi oil and a 4°C decrease in rumen incubation temperature for 7 days would reduce CH_4_ emissions and alter the ruminal microbial community. As such, the objective of this study was to evaluate the effect of pequi oil supplementation and incubation temperature on CH_4_ production and the ruminal microbiota in a Rusitec (rumen simulation technique) system.

## Materials and Methods

The donor cow was cared for in accordance with the guidelines of the German Animal Welfare Act approved by the Lower Saxony State Office for Consumer Protection and Food Safety (LAVES, approval number AZ 33.4-42505-04-13A373).

### Experimental Design and Treatments

*In vitro* ruminal fermentation was conducted using a Rusitec unit to evaluate the effect of incubation temperature and dietary fatty acids from pequi (*Caryocar brasiliense*) on the rumen microbiota and methane production. The experiment was a completely randomized design with two treatments and three replicates per treatment. The two treatments consisted of a control diet (CON, no pequi oil inclusion) and a diet supplemented with pequi oil (1.5 mL/day) which increased the dietary fat content to 6% (DM-basis).

### Plant Component

Pequi oil was selected to supplement the diets because of its high proportion of unsaturated fatty acids (Lima et al., 2007), which have been reported to be effective in reducing CH_4_ production without compromising diet digestibility and hence milk production in dairy cattle (Beauchemin et al., 2009).

### Substrate and Rumen Inoculum

The substrate consisted of hay:concentrate (66.7:33.3 w/w; **Table 1**). Hay substrate was prepared using an electric wool clipper with a 76 mm blade. The particle size distribution of hay material [% of DM] (Soofi-Siawash and Mathison, 1994) indicated that 6.5, 46.7, and 46.8% of plant DM was retained on sieves with aperture sizes of ≥4-mm, 1-mm, and ≤0.63-mm, respectively. The prepared substrates were weighed into nylon bags (11.5 cm × 6.5 cm, pore size 150 μm) for a total mass of 11 g of substrate (DM basis).

**Table 1:** Chemical composition of the substrates (%; w/w).

	Hay	Concentrate
Dry matter (DM)	89.74	89.68
Crude protein	10.7	17.0
Crude fat	2.1	3.1
Neutral detergent fiber	53.8	21.2
Ash	5.81	6.51

Rumen inoculum from a fistulated cow (Soliva et al., 2011; Soliva et al., 2015) was collected 2 hours after morning feeding (e.g., bag exchange). The diet fed to the donor cow and the substrate proportion (forage:concentrate ratio) used in the experiment were identical. Extracted rumen content was strained through four layers of cheesecloth to separate the solid and liquid portion and used as the initial inoculum.

### Chemical Composition

Feed was analyzed following AOAC (1995) methods for DM (Method 967.03), ash (Method 942), and ether extract (EE) content by extraction with petroleum ether using a Soxtherm Fat Extractor (Gerhardt Instruments, Königswinter, Germany; Method 920.39). Neutral detergent fibre (NDF) content was analyzed according to Van Soest et al. (1991) with the use of sodium sulfite and heat-stable a-amylase. Nitrogen (N) concentration was determined by the Kjeldahl method (Vapodest 20S, Gerhardt Instruments) with CP content calculated as N × 6.25.

### Rusitec Fermentation

One Rusitec unit with six fermentation vessels (800 mL capacity each) submerged in a water bath was set up for the experiment following the incubation procedure described by Czerkawski and Breckenridge (1977). The experiment was conducted over 28 days, with the first 7 days used as an adaptation period at 39°C incubation, followed by an additional 7 days at 39°C, then 7 days at 35°C and finally 7 days at 39°C. On the first day of the experiment 800 mL of rumen fluid along with individual nylon bags containing 10 g of solid digesta and 11 g of the diet (DM basis) were placed in each fermentation vessel within the unit (water bath). The fermentation vessels were immediately infused with modified McDougall’s buffer pH 7.35 with an osmolality of 293 mosm/L at a dilution rate of 30 mL/h. Pequi oil was added (1.5 mL) each day to the nylon bags prior to the bag exchange, according to the experimental design. Following the initial 24 h incubation period, the bag with the rumen digesta (rumen solids) was replaced with a nylon bag containing the substrate and after 48 hours the bags were replaced each day. Therefore, each fermentation vessel always had two bags present during the experiment (Avila-Stagno et al., 2014).

### Sample Collection and Analysis

#### Gas Production and Dry Matter (DM) Disappearance

Volumes of the effluent and total gas production were determined each morning before the nylon bags were exchanged. Accumulated effluent was collected in a 1 L glass flask and placed on ice to arrest microbial growth. Effluent output was measured daily during feed bag exchange. Total gas was collected in gas-tight bags (Plastigas, Linde AG, Munchen, Germany) and the volume of gas produced was measured using a drum-type meter (Ritter Apparatebau, Bochum, Germany) (Riede et al., 2013).

Throughout the experiment, pH and redox potential was measured daily during bag exchange using a Knick pH meter (digital pH meter 646, Knick, Berlin, Germany). DM disappearance was determined using the residue remaining in each nylon bag following 48 h of fermentation. Once removed, the nylon bags were washed with cold distilled water until the water ran clear. The clean bags were then dried at 55°C for 48 h (Narvaez et al., 2013; Li et al., 2014). The residue DM weight was then recorded and used for the calculation of DM disappearance.

#### Methane Production

Gas samples for methane analysis were taken using a 20 mL syringe and transferred into blood sample tubes (Venosafe type VF-109 SP, Terumo, Eschborn, Germany). The methane samples were analyzed using gas chromatography (GC 2014, Shimadzu Europa GmbH, Duisburg, Germany). Methane production was calculated by multiplying total gas volume by the percentage of CH_4_ in the gas sample and then corrected for standard temperature and pressure [0°C; 1013 hPa] (Riede et al., 2013).

#### Volatile Fatty Acids (VFA) and Ammonia Production

From d 1 to 28, before the replacement of substrate nylon bags, 5 mL of the effluent collected in the outflow flasks from each fermenter were taken and stored at –40°C. These samples were later used for volatile fatty acids (VFA) and ammonia (NH_3_) analyses. VFA concentration was analyzed by a gas chromatography system (model 5890 II, Hewlett Packard, Böblingen, Germany) equipped with a 1.8 m × 2 mm glass column packed with Chromosorb WAW (mesh 80/100) with 20% neopentyl glycol succinate and 2% orthophosphoric acid. Helium was used as a carrier gas with a flow rate of 25 mL/min. Injection port, detector and oven temperatures were 220, 250, and 130°C, respectively. VFA production was estimated by multiplying VFA concentration by the volume of effluent (Oeztuerk et al., 2005). For NH_3_ concentration analysis, 1 mL of sample was centrifuged at 4,600 × *g* for 10 min. From the supernatant, 50 μL were mixed with 5 mL phenol solution (106 mM phenol, 0.17 mM sodium nitroprusside dehydrate) and 5 mL sodium hypochlorite solution (1% v/v sodium hypochlorite; 125 mM NaOH) and kept at room temperature for 10 min. After an incubation step at 60°C for 10 min, NH_3_ concentration was determined photometrically at 546 nm in a spectrometer (DU 640, Beckman Coulter GmbH, Krefeld, Germany) using a NH_4_Cl standard solution (5 mM).

### DNA Extraction and Illumina Sequencing of the Archaeal and Bacterial 16S rRNA Gene

16S rRNA gene sequencing was used to analyze the archaeal and bacterial communities of the original rumen fluid and digesta (rumen solids), as well as the LAM (liquid-associated microbes) and SAM (solid-associated microbes) samples taken at the end of every experimental period (day 14, 21, and 28). Total genomic DNA was extracted from each sample using a QIAamp Fast DNA stool mini kit (Qiagen), according to manufacturer’s instructions, with a lysis temperature of 80°C. DNA yield and purity was quantified using an Eppendorf BioPhotometer 6131 (Eppendorf, Hamburg, Germany). Extracted DNA was stored at –20°C until use.

The 16S rRNA gene sequence libraries were generated using a two-step PCR protocol. The first PCR step amplified the V4 region of the 16S rRNA gene using the universal bacterial and archaeal primers 515-F (5′-GTGCCAGCMGCCGCGGTAA-3′) and 806-R (5′-GGACTACVSGGGTATCTAAT-3′) (Caporaso et al., 2011). The second PCR step was used to add a unique 10-bp barcode at the 5′ end of the amplicon as well to add Illumina adapters. All PCR amplification and sequencing steps were carried out at Genome Quebec (Montreal, QC, Canada). Briefly, the 16S rRNA gene amplicons were quantified using a Quant-iT PicoGreen dsDNA assay kit (Invitrogen, Burlington, ON, Canada), pooled in equimolar ratios and then purified with AMPure XP beads (Beckman Coulter, Mississauga, ON, Canada). The 16S rRNA gene amplicons were then sequenced using an Illumina MiSeq (2 × 250) and the MiSeq Reagent Kit v2 (Illumina, San Diego, CA, United States) according to the manufacturer’s instructions.

### Analysis of Archaeal and Bacterial 16S rRNA Gene Sequences

All 16S rRNA gene sequences were processed and analyzed within the QIIME software package v. 1.9.1 (Caporaso et al., 2010b). Paired-end reads were joined using fastq-join with a minimum overlap of 35 bp and a maximum percent difference of 15 (Aronesty, 2013). These joined sequences were then quality filtered with sequences truncated following three consecutive base calls of a Phred score of less than 25. Only those sequences containing 75% or more of the original sequence following truncation were retained for further analysis. Chimeric sequences were removed using the UCHIME algorithm (Edgar et al., 2011) implemented in USEARCH v. 6.1.544 (Edgar, 2010). Sequences were then clustered into operational taxonomic units (OTUs) at 97% similarity using an open reference OTU picking method and the SILVA database [v. 111] (Quast et al., 2013). Sequences that did not match OTUs in the SILVA database were clustered into OTUs using the *de novo* approach and USEARCH. The UCLUST consensus taxonomy assigner (Edgar, 2010) was used to assign taxonomy to OTUs using the SILVA database, with a minimum similarity of 0.8 and max accepts of 3. Representative sequences for the OTUs were aligned using PyNast (Caporaso et al., 2010a) and a phylogenetic tree was created using FastTree (Price et al., 2010). All OTUs containing fewer than 10 sequences as well as those OTUs classified as chloroplasts were excluded from further analysis.

Each sample was randomly subsampled to 23,000 sequences per sample to account for uneven sequencing depth. The bacterial and archaeal diversity in each sample was calculated within QIIME using the Shannon index (Shannon, 1948) and phylogenetic diversity (PD whole tree) (Faith, 1992). Unweighted UniFrac distances (Lozupone and Knight, 2005) were used to assess the bacterial and archaeal community structure (beta-diversity) of each treatment, incubation temperature and sample type (solid vs. liquid). The subsequent distance matrices were visualized as principal coordinate analysis (PCoA) plots using Emperor (Vazquez-Baeza et al., 2013). All 16S rRNA gene sequences were submitted to the NCBI Sequence Read Archive^1^ under bioproject accession SRP066918.

### Statistical Analysis

Fermentation data were analyzed using the MIXED procedure of SAS (SAS, Inc., 2015; SAS Online Doc 9.1.3). The model included the fixed effects of pequi oil, temperature and pequi oil × temperature interaction. Therefore, the individual fermenter was used as the experimental unit for statistical analysis. The minimum values of Akaike’s information criterion was used to select the covariance structure among compound symmetry, heterogeneous compound symmetry, autoregressive, heterogeneous autoregressive, Toeplitz, unstructured and banded for each parameter. Diversity metrics were analyzed using PROC MIXED in SAS 9.4 (SAS Inst., Inc., Cary, NC, United States) with fermenter as a random effect and pequi oil, temperature and interaction pequi oil × temperature as fixed effects. Treatment means were compared using the least squares mean linear hypothesis test (LSMEANS/DIFF). Significance was declared at *P* ≤ 0.05 and a trend was discussed when 0.05 < *P* ≤ 0.10.

The unweighted UniFrac distances were compared using ANOSIM (analysis of similarities) with 999 permutations within QIIME. Operational taxonomic units that were differentially abundant by sample type and treatment were identified using the G-test with a false discovery rate (FDR) *<* 0.05. Linear discriminant analysis effect size (LEfSe) was used to determine which phyla and genera were differentially abundant based on treatment, sample type and incubation temperature. The LEfSe uses the Kruskal–Wallis test to identify significantly different (*P* < 0.05) phyla and genera among sample groups and uses linear discriminant analysis (LDA) to estimate the effect size of each of these (Segata et al., 2011). A LDA score of 3.5 was used as the threshold for plotting differentially abundant genera.

## Results

### Effects of Incubation Temperature and Pequi Oil Supplementation on Fermentation Characteristics

Pequi oil supplementation had no effect on total gas, CH_4_ production or DM disappearance (*P* ≥ 0.58; **Table 2**). Decreasing the incubation temperature from 39°C to 35°C on d 15 had a negative effect on CH_4_ production reducing it from 80 mg/d to 34.3 mg/d. When incubation temperature was returned to 39°C on d 22, CH_4_ production was still lower (*P* < 0.01) than the initial 39°C incubation period (day 8–14). DM disappearance was greater (*P* < 0.01) during the 8–14 days incubation period and was diminished when the temperature was reduced to 35°C and through to the end of the experiment (**Table 2**).

**Table 2:** Effect of incubation temperature and pequi oil on methane production and dry matter disappearance (DMD).

	39°C		35°C		39°C					
	(Day 8–14)		(Day 15–21)		(Day 22–28)			*P*-value		
	Control	Pequi oil	Control	Pequi oil	Control	Pequi oil	SEM	Pequi oil	Temperature	Pequi oil × Temperature
Total gas (mL/d)	1851	1819	1505	1596	1597	1595	59.1	0.51	<0.01	0.41
CH_4_ (%)	4.85	5.75	2.49	3.89	2.01	1.10	1.188	0.70	0.01	0.50
CH_4_ (mL/d)	109.5	114.8	44.0	52.1	44.5	37.5	10.65	0.81	<0.01	0.75
CH_4_ (mg/d)	78.2	82.0	31.4	37.2	31.8	26.8	7.60	0.81	<0.01	0.75
DMD (%)	68.9	71.5	61.6	63.0	63.6	64.3	2.52	0.58	<0.01	0.59

*Mean values with standard errors.*

There was a tendency (*P* = 0.10) toward lower pH at 35°C compared to the last period at 39°C (6.76 vs. 6.78) and pequi oil supplementation decreased (*P* = 0.03) pH compared to the control (6.74 vs. 6.78). The redox potential was not affected by pequi oil addition but was lower (*P* < 0.01) during the 22–28 days period (39°C) compared to other time periods. An interaction between pequi oil supplementation and incubation temperature was observed for total VFA, acetate, propionate and iso-valerate production and the acetate:propionate ratio (*P* ≤ 0.05; **Table 3**). Supplementation with pequi oil only increased (*P* = 0.03) total VFA production during the 22–28 days fermentation period. Pequi oil increased (*P* ≤ 0.01) acetate and propionate production, following the initial adaptation period and throughout the experiment. The acetate:propionate ratio was decreased (*P* = 0.04) with the addition of pequi oil following the adaptation period (day 1–7). Ammonia production was also lower (*P* < 0.01) at the 35°C (day 15–22) incubation temperature compared to 39°C, but was not affected by pequi oil supplementation.

**Table 3:** Effect of incubation temperature and pequi oil on pH, redox potential and fermentation characteristics.

	39°C		35°C		39°C					
	(Day 8–14)		(Day 15–21)		(Day 22–28)			*P*-value		
	Control	Pequi oil	Control	Pequi oil	Control	Pequi oil	SEM	Pequi oil	Temperature	Pequi oil × Temperature
pH	6.8	6.7	6.8	6.7	6.8	6.8	0.02	0.03	0.10	0.51
Redox potential (mV)	–177.0	–185.3	–190.4	–197.0	–235.0	–235.6	11.27	0.64	<0.01	0.97
Total VFA (mmol/d)	32.6	38.3	31.6	34.2	33.0b	36.8a	1.77	0.03	<0.01	0.03
Acetate (mmol/d)	16.8b	19.5a	16.0	17.1	17.0b	19.1a	0.43	0.01	<0.01	0.02
Propionate (mmol/d)	5.9b	7.6a	5.9b	6.9a	6.5b	7.6a	0.16	<0.01	<0.01	<0.01
Butyrate (mmol/d)	5.5	6.5	5.4	5.6	6.1	6.2	0.25	0.47	<0.01	0.13
Valerate (mmol/d)	2.4	2.6	2.0	2.1	2.2	2.3	0.15	0.57	<0.01	0.76
Iso-valerate (mmol/d)	0.79b	1.17a	0.94	1.19	1.01b	1.19a	0.017	0.01	<0.01	0.03
Acetate:propionate	2.85a	2.57b	2.71a	2.48b	2.62a	2.51b	0.080	<0.01	<0.01	0.04
Ammonia (mmol/d)	6.9	7.2	5.5	5.9	6.5	6.5	0.09	0.31	<0.01	0.19

*Different lowercase letters in rows indicate significantly different means (*P* < 0.05).*

### Rumen Microbiota in the Rusitec System

**FIGURE 1:**
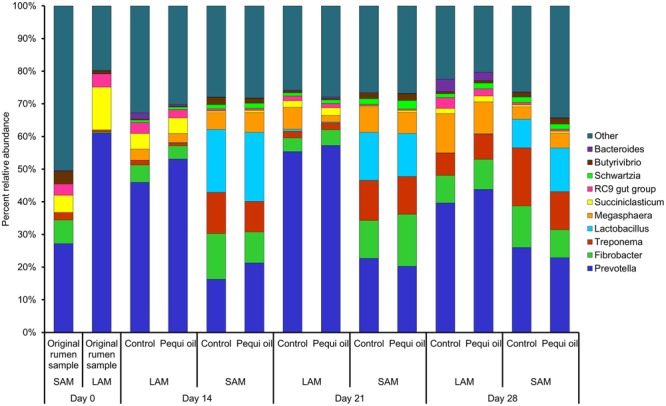
Classification of 16S rRNA gene sequences at the genus level for each individual sampling day, incubation temperature, sample type and treatment. Only the ten most relatively abundant genera are displayed (*N* = 3 for all samples). LAM = liquid-associated microbes, SAM = solid-associated microbes.

There was a total of 1,400,917 16S rRNA gene sequences with an average length of 262 bp following quality filtering. These sequences were clustered into 4,384 OTUs and classified into 20 different phyla and 89 distinct genera. The most relatively abundant phyla among all samples were: *Bacteroidetes* (45.8%), *Firmicutes* (25.9%), *Fibrobacteres* (8.8%), *Spirochaetes* (7.8%), *Actinobacteria* (5.9%) and *Euryarchaeota* (1.7%). The most relatively abundant genus was *Prevotella* (35.8%), followed by *Fibrobacter* (8.8%), *Treponema* (7.6%), *Lactobacillus* (7.2%) and *Megasphaera* (5.6%) (**Figure 1**). In terms of methanogens, overall there were three genera identified, all with a relative abundance of less than 0.1%: *Methanobrevibacter* (0.09%), *Methanomicrobium* (0.01%), and *Methanosphaera* (0.0008%). However, in the original rumen solid sample, *Methanobrevibacter* comprised 14.6% of the total microbial population.

### Effect of Pequi Oil, Sample Type, and Incubation Temperature on Archaeal and Bacterial Diversity and Microbiota Structure

**FIGURE 2:**
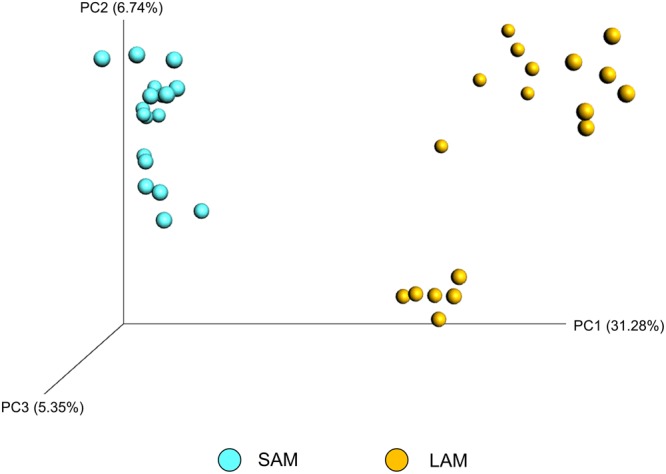
Principal coordinate analysis (PCoA) of the unweighted UniFrac distances for SAM and LAM samples. The percent variation explained by each principal coordinate is indicated on the axes. Original rumen samples are excluded. LAM, liquid-associated microbes; SAM, solid-associated microbes. *N* = 18 for both sample types.

**FIGURE 3:**
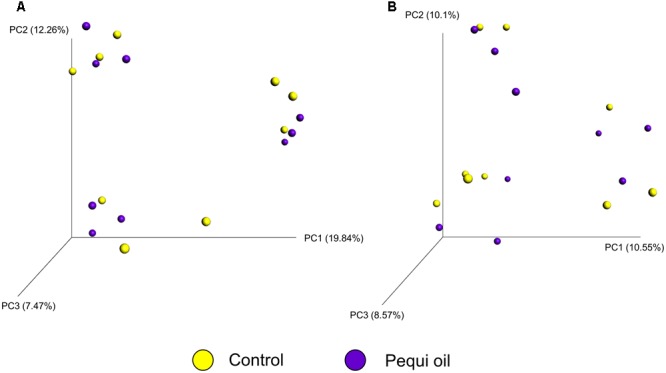
Principal coordinate analysis (PCoA) of the unweighted UniFrac distances for the effect of pequi oil supplementation on **(A)** LAM and **(B)** SAM samples. The percent variation explained by each principal coordinate is indicated on the axes. No rumen samples included. LAM, liquid-associated microbes; SAM, Solid-associated microbes.

**FIGURE 4:**
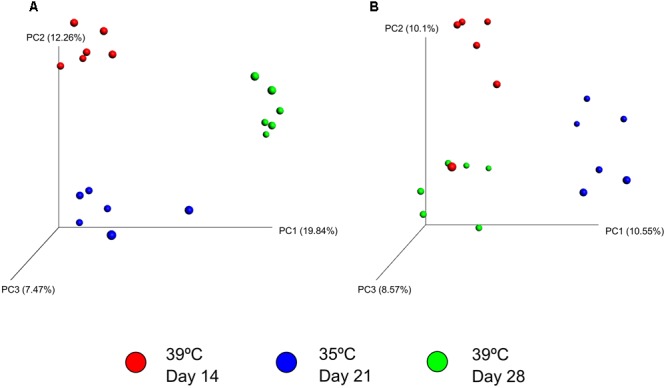
Principal coordinate analysis (PCoA) of the unweighted UniFrac distances for **(A)** LAM samples and **(B)** SAM samples for each sampling day and incubation temperature. The percent variation explained by each principal coordinate is indicated on the axes. Original rumen samples are excluded. LAM, liquid-associated microbes; SAM, solid-associated microbes. *N* = 6 for each sample type, sampling time and incubation time.

There was greater archaeal and bacterial diversity and richness in the SAM samples compared with the LAM samples based on the alpha-diversity metrics listed in **Table 4** (*P* < 0.05). Alpha-diversity analysis also suggested that a temperature effect was present only in the LAM samples as LAM samples incubated at 39°C had a higher Shannon diversity value and greater phylogenetic diversity (*P* < 0.01). The addition of pequi oil had no effect on any of the diversity or richness metrics for either LAM or SAM samples. Unweighted UniFrac distances were used to determine the effect that pequi oil and incubation temperature had on the archaeal and bacterial community structure. Similar to the within-sample diversity analysis, ANOSIM of unweighted UniFrac distances demonstrated that sample type (LAM vs. SAM) was the most important factor in determining the archaeal and bacterial community structure (**Figure 2**; *R*-value = 0.994; *P* < 0.01). Pequi oil did not have any significant effect on the archaeal and bacterial community structure among either the LAM (**Figure 3A**; *R*-value = –0.010; *P* = 0.44) or SAM (**Figure 3B**; *R*-value = –0.025; *P* = 0.59) samples. In contrast, there was a strong temperature and time effect present in both the LAM (**Figure 4A**, *R*-value = 0.395; *P* < 0.01) and SAM (**Figure 4B**; *R*-value = 0.410; *P* < 0.01) samples. However, it should be noted that the microbial community on day 28 did not return to a completely similar state as it was on day 14, when the incubation temperature was also 39°C.

**Table 4:** Richness and diversity measures for each sampling day, incubation temperature, sample type and treatment (*N* = 3).

	39°C		35°C		39°C					
	(Day 8–14)		(Day 15–21)		(Day 22–28)			*P*-value		
	Control	Pequi oil	Control	Pequi oil	Control	Pequi oil	SEM	Pequi oil	Temperature	Pequi oil × Temperature
**LAM**										
Shannon index	4.44	4.38	4.12	4.19	4.51	4.47	0.084	0.87	<0.01	0.75
PD whole tree	81.6	83.9	81.0	78.2	89.6	89.3	1.74	0.85	<0.01	0.38
Observed OTUs	1292	1339	1323	1242	1465	1506	51.8	0.96	<0.01	0.41
**SAM**										
Shannon index	4.49	4.41	4.52	4.74	4.60	4.51	0.103	0.87	0.26	0.28
PD whole tree	85.7	85.4	83.1	87.4	88.1	86.3	1.38	0.53	0.35	0.11
Observed OTUs	1498	1488	1481	1624	1529	1473	38.0	0.42	0.27	0.05

*LAM = liquid-associated microbes, SAM = solid-associated microbes.*

### Differentially Abundant OTUs and Taxa between Sample Types, Incubation Temperatures, and Treatment Groups

**FIGURE 5:**
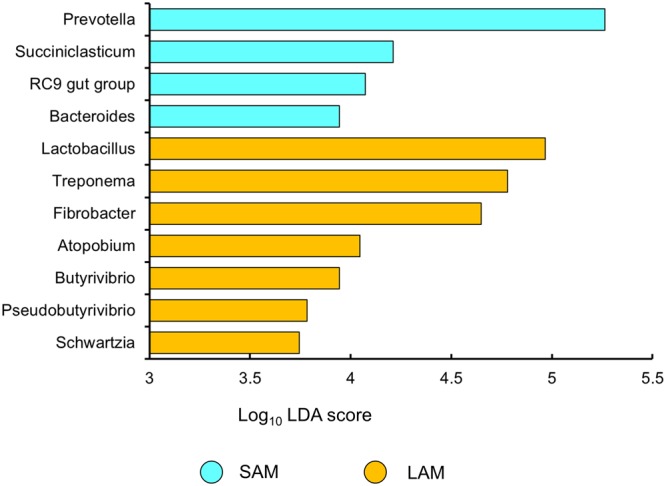
Differentially abundant genera between LAM and SAM samples as determined using linear discriminant analysis effect size (LEfSe) analysis. Only genera with a LDA score greater than 3.5 and an overall relative abundance of greater than 0.01% were included in the analysis.

Differentially abundant genera between pequi oil and control samples were identified using LEfSe. *Anaerovibrio* was found to be enriched in both the LAM and SAM samples under the pequi oil treatment compared with the control. The only other differentially abundant genus in either treatment group was *Anaerovorax* which was enriched in the control group of SAM samples (LDA score > 3.5; *P* < 0.05). When differences between LAM and SAM samples were assessed, a total of 11 genera were enriched in one of the two sample types (**Figure 5**; LDA score > 3.5). Of these genera, *Prevotella* was most notably enriched in the SAM samples and *Lactobacillus* in the LAM samples. Only three genera were enriched at one of the two incubation temperatures; *Bacteroides* and the RC9 gut group at 39°C and *Streptococcus* at 35°C (LDA score > 3.5; *P* < 0.05).

Excluding the original rumen samples, there were 194 OTUs that were differentially abundant (FDR < 0.05) between solid (84 OTUs) and liquid (110 OTUs) samples (Supplementary Table S1). There were also 57 OTUs that were differentially abundant between the samples incubated at 35°C and 39°C, with 27 more abundant at 35°C and 30 at 35°C (Supplementary Table S2). The most relatively abundant of these OTUs for both temperatures were classified as *Prevotella* spp. Only 20 OTUs were differentially abundant between the pequi oil (13 OTUs) and control (7 OTUs) samples (Supplementary Table S3).

## Discussion

One of the strategies for lowering enteric methane emissions in ruminants has been the supplementation of diets with fatty acids due to their ability to decrease available organic matter fermented in the rumen, reduce methanogen activity and protozoal populations (Johnson and Johnson, 1995). In the present study, we evaluated the combined effect of pequi oil supplementation and incubation temperature shifts in a Rusitec system. Pequi oil comprised 6% of dietary fat content (DM-basis), as it has been recommended that fat inclusion in diets for the purpose of mitigating methane production should not exceed 6-7% due to the depression in DM intake (Johnson and Johnson, 1995). Despite the high content of the unsaturated fatty acid C18:1 in pequi oil (Segall et al., 2006), we did not find an inhibitory effect on methane production. In contrast, Beauchemin et al. (2009) reported a daily reduction of 13% in CH_4_ production when diets were supplemented with crushed oilseeds (3.7% supplementation) that were high in fatty acids (C18:1).

Our findings on methane production are more in accordance with the results of Johnson et al. (2002), who found that dietary fat supplementation from oilseeds (cotton and canola seeds) did not affect CH_4_ emissions *in vivo* at inclusion concentrations of 2.3, 4.0, and 5.6%. However, information regarding methane reduction and monounsaturated fatty acids (e.g., C18:1) is more limited when compared with polyunsaturated fatty acids such as linoleic and linolenic acid (Martin et al., 2010). In the current study, we found that supplementation with pequi oil had a positive effect on propionate production and a negative effect on the acetate:propionate ratio (**Table 3**) following the adaptation period (day 1–7; data not shown). However, these effects were influenced by an interaction between supplementation and incubation temperature, with greater propionate production at 39°C and a lower acetate:propionate ratio at 35°C, although propionate production was greater compared with the control diet at 35°C.

The effect that lipids have on methane abatement is of interest due to their potential to decrease methanogenesis through inhibition of protozoa and increased production of propionic acid (Johnson and Johnson, 1995). However, pequi oil supplementation did not alter the archaeal community in the present study. The lack of effect of pequi oil could be related to the relatively small concentration of pequi oil used in relation to the total volume of the fermenter (1.5 mL pequi oil/820 mL fermenter). When expressed in terms of incubated substrate DM, pequi oil comprised 6% of dietary fat content. However, it represents only 0.18% of the total fermenter volume and therefore, this may account for the differences observed in the current study in relation to CH_4_ production compared to earlier work in our lab using batch culture and pequi oil (Unpublished data).

Environmental temperature has been associated with methane production and changes in feed digestibility (Shibata and Mukai, 1979; Nonaka et al., 2008; Shibata and Terada, 2010). In the current work, we observed that CH_4_ production was negatively affected by incubation temperature. Decreasing the fermentation temperature to 35°C from 39°C reduced CH_4_ production (mg/d) by 43%. Similarly, DM digestibility was also reduced at 35°C. To date, relatively few *in vitro* studies have evaluated the effect of temperature on fermentation parameters and methane production. Bhatta et al. (2006), for example, evaluated the effect of two different incubation temperatures in a Rusitec and concluded that a minor increase in temperature (39°C vs. 41°C) did not have any significant effect on methane production. These authors also reported that pH was the most important factor affecting microbial production of VFA and CH_4_, suggesting that altering pH might be a way to optimize VFA production and reduce CH_4_ production (Bhatta et al., 2006). Decreasing the incubation temperature to 35°C for 7 days and returning to normal rumen temperature of 39°C is a potentially novel way to affect CH_4_ production. In justification for this experiment, the reduction of the rumen temperature *in vivo* could be similarly achieved through the introduction of a specific device into the rumen which could adjust rumen temperature. This would allow for further evaluation of decreased rumen temperature on rumen fermentation and production parameters.

High-throughput sequencing of the archaeal and bacterial 16S rRNA gene revealed that the addition of pequi oil to the Rusitec system did not alter the ruminal microbiota. With the exception of the original rumen SAM sample, the relative abundance of methanogens remained low throughout the study (1.4% overall). *Methanobrevibacter* spp. were the most relatively abundant methanogens, a finding that is in accordance with Henderson et al. (2015). These authors reported that members of the *Methanobrevibacter* were widely distributed in the microbiota of several different ruminant species and accounted for 74% of all ruminal archaeal genera. Among other methanogens, only *Methanosphaera* and *Methanomicrobium* were identified, an expected finding given that the rumen archaeal microbiota is much less rich and diverse compared to the bacterial microbiota (Henderson et al., 2015).

We observed increased production of propionate and reduced acetate:propionate ratio in the present study. It is possible that this result was due to the high relative abundance of *Prevotella* in this experiment, as *Prevotella* spp. have been reported to be propionate producers (Russell and Hespell, 1981; McCabe et al., 2015) and members of this genus have shown clear dominance in the rumen (Stevenson and Weimer, 2007; Henderson et al., 2013). Although pequi oil supplementation did not have any effect on the structure of the archaeal and bacterial microbiota in the Rusitec, the genus *Anaerovibrio* was found to be enriched in the pequi oil-supplemented samples. *Anaerovibrio* has been reported to have an important role in the ruminal metabolism of lipids, producing substrates used for propionate synthesis (Jouany et al., 2007; Jenkins et al., 2008).

The archaeal and bacterial richness and diversity indices suggested a temperature effect, but only for the LAM samples, with lower diversity and richness observed in LAM samples incubated at 35°C (day 14 to 21). When the incubation temperature was returned to 39°C during days 21 to 28, the richness and phylogenetic diversity actually increased in comparison with the original 39°C incubation period. In addition, incubation temperature did not affect the diversity of the SAM samples, which may explain why methanogen populations were also not affected by incubation temperature. However, the reduction in methane production which continued when the temperature was returned to the original incubation temperature of 39°C (day 22–28), suggests that methanogen activity was affected but not the overall microbial community. In addition, it is possible that using a PCR primer pair that is more selective and sensitive for methanogens may provide better resolution of the ruminal methanogenic community.

## Conclusion

We determined that a 4°C decrease in temperature resulted in a 57% reduction of methane production and also affected microbial diversity, but only for LAM samples. This could suggest that specific methanogens within the LAM samples have an important role in methane production. Although the supplementation with pequi oil in this study did not affect methane production or microbial diversity, propionate production was enhanced. The microbiota of both the LAM and SAM samples were also strongly affected by temperature/sampling time. Further *in vivo* studies manipulating the rumen temperature are advised to verify its effect on CH_4_ emissions and/or its effects on the microbiome.

## Author Contributions

Study design: AC, GB, AD. Conducting RUSITEC study: AD, AC. Lab analysis: AD, KK, DH, TA, GB, AC. DNA extraction: AD, KK. Bioinformatics: DH, AD. Writing the manuscript: AD, DH, TA, GB, AC.

## Conflict of Interest Statement

The authors declare that the research was conducted in the absence of any commercial or financial relationships that could be construed as a potential conflict of interest.

## Footnotes

1 http://www.ncbi.nlm.nih.gov/sra/
